# Systematic review and meta‐analysis of vascular endothelial growth factor as a biomarker for malignant pleural effusions

**DOI:** 10.14814/phy2.12978

**Published:** 2016-12-27

**Authors:** Eleftheria Fafliora, Chrissi Hatzoglou, Konstantinos I. Gourgoulianis, Sotirios G. Zarogiannis

**Affiliations:** ^1^ Primary Health CareFaculty of MedicineUniversity of ThessalyLarissaGreece; ^2^ Department of PhysiologyFaculty of MedicineUniversity of ThessalyLarissaGreece; ^3^ Department of Respiratory MedicineFaculty of MedicineUniversity of ThessalyLarissaGreece

**Keywords:** Benign pleural effusion, biomarkers, malignant pleural effusion, meta‐analysis, vascular endothelial growth factor

## Abstract

Conventional methods may fail to identify the cause of pleural effusion (PE), thus establishing reliable biomarkers is deemed necessary. This study aimed at examining the role of vascular endothelial growth factor (VEGF) as a biomarker in the differentiation between malignant and benign PEs in adults. A comprehensive literature search in PubMed (Medline), Scopus (ELSEVIER), and Cochrane Central Register of Controlled Trials (CENTRAL) databases was conducted using keywords. We included studies that evaluated pleural and/or serum levels of VEGF among patients presenting with undiagnosed PE and the association between these levels and the final diagnosis. We performed a meta‐analysis to calculate the summary effect using the random effects model. Statistical analysis was performed with the statistical package for meta‐analysis Comprehensive Meta‐Analysis. Twenty studies were included in the systematic review, while 11 of them in the meta‐analysis. Pleural fluid VEGF levels among patients with malignant PE were increased by 1.93 ng/mL as compared to patients with benign PE (95% CI: 1.32–2.54, *Q* = 173, df (*Q*): 10, *I* ^2^ = 94.2%, *P* < 0.05). Serum VEGF levels among patients with malignant PE were increased respectively by 1.90 ng/mL (95% CI: 0.93–2.88, *Q* = 182, df (*Q*): 6, *I* ^2^ = 96.7%, *P* < 0.05). This study showed that malignant PEs were associated with higher levels of both pleural fluid and serum VEGF. VEGF appears to represent a promising biomarker for the differential diagnosis between benign and malignant PEs.

## Introduction

The annual incidence of pleural effusion (PE) in the United States alone is estimated to be 1.5 million (Janda and Swiston 2010). PE is the result of a variety of medical disorders and therefore the diagnosis of the exact cause can be challenging. Congestive heart failure, pneumonia, and malignancy are the most common causes of PE with an annual estimated incidence of 500,000, 300,000, and 200,000 cases, respectively (Porcel and Light 2006).

The initial investigation of a patient with suspected PE includes clinical history, clinical examination, and a chest X‐ray, whereas in most cases, thoracentesis with aspiration of pleural fluid is required (Porcel and Light 2006; British Thoracic Society 2010). However, the sensitivity of thoracocentesis and cytological, histological and biochemical examinations are reported to be only 40–70% (Chen et al. 2015). Concerning the Light criteria, used in the differentiation between exudates and transudates, they are extremely sensitive in identifying exudates, but they lack specificity (Porcel 2013). Furthermore, only 50–70% of patients with malignant PE (MPE) can be diagnosed by cytological examination of the pleural fluid (Chen et al. 2015).

Since PE can result from a variety of conditions, the establishment of more disease‐specific methods in order to contribute to the value of the existing diagnostic tests and thus enable faster and more accurate diagnosis of the cause of PE is deemed necessary. For this purpose, a variety of biomarkers has been examined. Increased levels of NT‐proBNP have been positively correlated with congestive heart failure as a cause of the PE (Porcel and Light 2006; Porcel 2013). Furthermore, CRP levels have been reported to be higher in parapneumonic PEs (Hassan et al. 2012; Zou et al. 2012).

Moreover, many molecules have been studied in order to contribute to the diagnosis of suspected MPE. Survivin or several tumor markers have been associated with the presence of malignancy as the cause of PE, however, the results are yet inconclusive (Liang et al. 2008; Hassan et al. 2012; Chen et al. 2015). Among the various markers found in MPE, the role of the vascular endothelial growth factor (VEGF) has been examined in several studies (Thickett et al. 1999; Lim et al. 2000; Ishimoto et al. 2002). VEGF has been shown to increase the permeability of the pleural mesothelium and vasculature and to be an important angiogenic factor contributing to pleural fluid accumulation (Cheng et al. 1999; Mohammed et al. 2001; Bradshaw et al. 2013; Peppa et al. 2014). It is important to note that VEGF receptors are present in the mesothelial cells of healthy and diseased human pleurae and thus VEGF is detected in both MPE and benign PE (BPE) (Cheng et al. 1999, 2000). However, it is reported that pleural fluid VEGF (PF‐VEGF) levels in patients with PE due to malignancies are higher than those due to benign diseases, rendering PF‐VEGF into a promising biomarker to aid in the differentiation between the two (Cheng et al. 1999; Kishiro et al. 2002; Bradshaw et al. 2013).

The aim of this study was to collect, examine, and compare all available data regarding PF‐VEGF and serum VEGF (S‐VEGF) levels among adult patients with MPE and BPE, so that the role of VEGF as a biomarker would be adequately substantiated.

## Materials and Methods

### Search strategy

We followed the guidelines of the Preferred Reporting Items for Systematic Reviews and Meta‐Analyses (PRISMA) Statement (Moher et al. 2009). The PRISMA Statement consists of a 27‐item checklist and a four‐phase flow diagram and its aim is to improve the reporting of systematic reviews and meta‐analyses. We conducted a comprehensive literature search of PubMed (Medline), Scopus (ELSEVIER) and Cochrane Central Register of Controlled Trials (CENTRAL) from their inception through to July 2015.

The following keywords were used for the search: vascular endothelial growth factor, pleural effusion, benign pleural effusion, malignant pleural effusion, diagnosis, serum, tuberculous pleurisy, heart failure, cancer, lung cancer, metastatic cancer. Furthermore, we utilized the PubMed Advanced Search Builder and the Advanced Search form in Scopus as well as relevant search filters. We used the Boolean operator “AND” in order to limit the retrieved articles to those that address the condition of interest and the outcomes to be evaluated, that is studies that compared VEGF levels between patients with BPE and MPE (Higgins and Green 2008). The search strategy document is provided in detail in the Appendix.

### Study selection

The initial search included all studies independent of their design that examined VEGF levels in the pleural fluid and/or serum from patients presenting with undiagnosed PE. The following exclusion criteria were applied: non‐relevant studies, non‐English studies (due to lack of funding), non‐human studies, pediatric population, small sample size (<10 patients). The references of the retrieved studies were manually searched in order to identify additional studies. A reference management software package was used in order to remove duplicate publications (EndNote^®^ X5; © Thomson Reuters, New York, NY). We did not perform a search for unpublished literature.

In all steps, the studies were independently reviewed by two authors (EF, SGZ) and the eligibility criteria were assessed. Disagreements between the two authors were resolved through consensus.

### Data extraction

One author (EF) extracted the data manually and recorded them in prespecified tables that were created using the Microsoft Office Word 2007. A second author (SGZ) confirmed the validity of the data, while discrepancies were solved through consensus. The data that were extracted from the studies included: authors names, publication year, country where the study was performed, study design, sample size, group of patients, diagnostic procedures and laboratory sample analysis methods, effect size and outcome (mean or median values of VEGF levels of different patient groups and comparisons between the groups).

### Assessment of methodological quality

The methodological quality of the included studies was assessed using the Newcastle–Ottawa scale for nonrandomized studies (Wells et al. 2013). The scale is based on a “star system” in which a study is judged on three broad perspectives: the selection of the study groups; the comparability of the groups; and the ascertainment of either the exposure or the outcome of interest. The maximum number of stars that can be appointed to a study is nine. Since the threshold score distinguishing between “fair” and “poor” quality studies has not yet been identified, our decision regarding scoring was based on relevant literature review. Therefore, we decided to classify studies as low quality if they received seven stars or less and as fair quality if they received eight or nine stars.

### Statistical analysis

In order to address the aim of our study, the patients were grouped as having either MPE or BPE. When the studies grouped their patients in several smaller groups based on the diagnosis of the cause of the PE, the appropriate ones were merged in order to create a single pair‐wise comparison as described before. The combining of the groups was conducted using the relevant formulae provided by the *Cochrane handbook for systematic reviews of interventions* (Higgins and Green 2008).

We used the random effects model in order to pool the effect sizes and calculate the standardized mean difference (SMD) and the 95% confidence interval from the mean and standard deviation of the PF‐VEGF and S‐VEGF levels among patients with MPE and BPE. The random effects model was chosen since we did not expect that all the identified studies would be functionally equivalent and that they would share a common effect size. We then proceeded to the identification of heterogeneity through the computation of the *Q* = statistic (with *P* < 0.10 indicating heterogeneity). Also, we quantified the degree of heterogeneity through the computation of the *I* ^2^ statistic (with values of 25%, 50%, and 75% indicating low, moderate, and high heterogeneity, respectively) (Borenstein et al. 2009).

The presence of publication bias was assessed initially by inspection of the funnel plot and confirmation or not of the visual impression by the Egger's test (with *P* < 0.05 indicating the presence of publication bias). We further examined whether the computed summary effect is solely an artifact of bias through the Rosenthal Fail‐safe *n*, which calculates the number of additional studies that would be needed in order to change the results of the meta‐analysis. Finally, we performed the Duval and Tweedie's Trim and Fill test in order to examine the impact that the publication bias might have, by calculating the best estimate of an “unbiased” effect size (Borenstein et al. 2009).

A *P* value of less than 0.05 (two‐tailed) was set as the threshold indicating a statistically significant result. We used the statistical package Comprehensive Meta‐Analysis, version 2.2.064 (Biostat, Englewood, CO) for our analyses.

## Results

### Study selection and characteristics of the included studies

**Figure 1:**
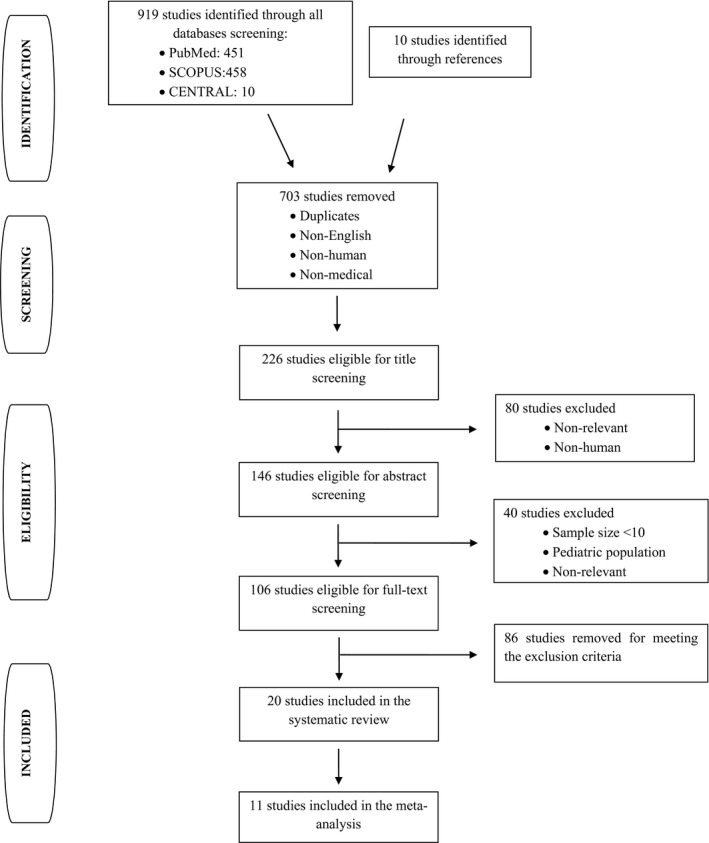
Flow diagram of the search strategy.

The literature search through all three databases as well as through the references of the retrieved studies returned 929 studies in total. Two‐hundred and twenty‐six articles were eligible for title screening. Eighty studies were excluded after title screening and additionally 40 studies after abstract screening due to meeting the exclusion criteria. One hundred and six studies were considered to be eligible for full‐text screening. Finally, 20 studies met the inclusion criteria and were included in the systematic review (Thickett et al. 1999; Yanagawa et al. 1999; Lim et al. 2000; Ishimoto et al. 2002; Momi et al. 2002; Hamed et al. 2004; Jin et al. 2004; Sack et al. 2005; Daniil et al. 2007; Shu et al. 2007; Tomimoto et al. 2007; Xue et al. 2007; Duysinx et al. 2008; Economidou et al. 2008; Zhou et al. 2009; Fiorelli et al. 2011; Hirayama et al. 2011; Qian et al. 2012; Zhang et al. 2012; Lieser et al. 2013), whereas 11 of them in the meta‐analysis (Momi et al. 2002; Hamed et al. 2004; Jin et al. 2004; Sack et al. 2005; Daniil et al. 2007; Shu et al. 2007; Xue et al. 2007; Zhou et al. 2009; Hirayama et al. 2011; Qian et al. 2012; Zhang et al. 2012). The flow diagram of the search strategy, according to the PRISMA guidelines, is presented in Figure 1.

All studies included in the systematic review were cohort studies, except one which was a case–control study (Hamed et al. 2004). A total number of 1696 patients presenting in health units with undiagnosed PE was assessed and grouped according to the cause of the PE. All studies evaluated PF‐VEGF levels, whereas 10 of them also evaluated the S‐VEGF levels. PF‐VEGF or S‐VEGF levels were compared among the different groups of patients. Half of the studies were deemed to be of low quality, mainly due to lack of comparability of cohorts. The characteristics of the included studies are presented in Table 1.

**Table 1:** Characteristics of the studies included in the systematic review

References, country	Study design	Study participants	Intervention‐method	Outcomes	Quality assessment
Thickett et al. (1999), United Kingdom	Cohort study	78 patients with undiagnosed PE	• Diagnostic thoracocentesis • ELISA for VEGF levels measurement	Comparison of pleural fluid VEGF levels between patients with MPE and BPE	• Selection **** • Comparability ‐ • Outcome *** *Overall* Low quality
Yanagawa et al. (1999), Japan	Cohort study	111 patients with undiagnosed PE	• Diagnostic thoracocentesis • ELISA for VEGF levels measurement	1 Comparison of pleural fluid VEGF levels between patients with exudative and transudative PE 2 Comparison of pleural fluid VEGF levels between patients with exudative PE and PE due to lung cancer	• Selection ** • Comparability ‐ • Outcome *** *Overall* Low quality
Lim et al. (2000), Korea	Cohort study	28 patients with undiagnosed PE	• Diagnostic thoracocentesis • ELISA for VEGF levels measurement	Comparison of pleural fluid VEGF levels between patients with MPE and tuberculous PE	• Selection **** • Comparability ** • Outcome *** *Overall* Fair quality
Ishimoto et al. (2002), Japan	Prospective cohort study	40 patients with undiagnosed PE	• Diagnostic thoracocentesis • Simultaneous blood sampling • ELISA for VEGF levels measurement	Comparison of pleural fluid and serum VEGF levels between patients with MPE and BPE	• Selection **** • Comparability ‐ • Outcome *** *Overall* Low quality
Momi et al. (2002), Japan	Cohort study	127 patients with undiagnosed PE	• Diagnostic thoracocentesis • Simultaneous blood sampling • ELISA for VEGF levels measurement	Comparison of pleural fluid and serum VEGF levels among patients with MPE, parapneumonic PE, PE due to congestive heart failure and tuberculous PE	• Selection **** • Comparability * • Outcome *** *Overall* Fair quality
Hamed et al. (2004), Egypt	Case–control study	30 patients with exudative PE and 20 controls (10 healthy, 10 with PE due to heart failure)	• Diagnostic thoracocentesis • Simultaneous blood sampling • ELISA for VEGF levels measurement	Comparison of pleural fluid and serum VEGF levels between case and controls	• Selection ** • Comparability * • Outcome ** *Overall* Low quality
Jin et al. (2004), South Korea	Cohort study	83 patients with undiagnosed PE	• Diagnostic thoracocentesis • Simultaneous blood sampling • ELISA for VEGF levels measurement	Comparison of pleural fluid and serum VEGF levels among patients with MPE, BPE and tuberculous PE	• Selection **** • Comparability ** • Outcome *** *Overall* Fair quality
Sack et al. (2005), China	Cohort study	214 patients with undiagnosed PE	• Diagnostic thoracocentesis • Simultaneous blood sampling • ELISA for VEGF levels measurement	Comparison of pleural fluid and serum VEGF levels among patients with PE due to lung cancer, PE due to other malignancies, tuberculous PE, inflammatory PE, PE due to congestive heart failure	• Selection **** • Comparability ** • Outcome *** *Overall* Fair quality
Daniil et al. (2007), Greece	Prospective cohort study	72 patients with undiagnosed PE	• Diagnostic thoracocentesis • ELISA for VEGF levels measurement	Comparison of pleural fluid VEGF levels among patients with MPE, tuberculous PE and parapneumonic PE	• Selection **** • Comparability ‐ • Outcome *** *Overall* Low quality
Shu et al. (2007), China	Prospective cohort study	81 patients with undiagnosed PE	• Diagnostic thoracocentesis • Simultaneous blood sampling • ELISA for VEGF levels measurement	Comparison of pleural fluid and serum VEGF levels between patients with MPE and BPE	• Selection **** • Comparability ** • Outcome *** *Overall* Fair quality
Tomimoto et al. (2007), Japan	Cohort study	42 patients with undiagnosed PE	• Diagnostic thoracocentesis • ELISA for VEGF levels measurement	Comparison of pleural fluid VEGF levels among patients with PE due to lung cancer, PE due to other malignancies, tuberculous PE, and PE due to congestive heart failure	• Selection **** • Comparability ‐ • Outcome *** *Overall* Low quality
Xue et al. (2007), China	Cohort study	87 patients with undiagnosed PE	• Diagnostic thoracocentesis • ELISA for VEGF levels measurement	Comparison of pleural fluid VEGF levels between patients with MPE and tuberculous PE	• Selection **** • Comparability ‐ • Outcome *** *Overall* Low quality
Duysinx et al. (2008), Belgium	Cohort study	106 patients with undiagnosed PE	• Diagnostic thoracocentesis • ELISA for VEGF levels measurement	Comparison of pleural fluid VEGF levels between patients with MPE and BPE	• Selection **** • Comparability * • Outcome *** *Overall* Fair quality
Economidou et al. (2008), Greece	Cohort study	67 patients with undiagnosed PE	• Diagnostic thoracocentesis • Simultaneous blood sampling • ELISA for VEGF levels measurement	1 Comparison of pleural fluid and serum VEGF levels between patients with exudative PE and PE due to congestive heart failure 2 Comparison of pleural fluid and serum VEGF levels among patients with MPE, inflammatory PE and undiagnosed PE	• Selection **** • Comparability ‐ • Outcome *** *Overall* Low quality
Zou et al. (2012), China	Cohort study	126 patients with undiagnosed PE	• Diagnostic thoracocentesis • ELISA for VEGF levels measurement	Comparison of pleural fluid VEGF levels between patients with MPE and BPE	• Selection **** • Comparability * • Outcome *** *Overall* Fair quality
Fiorelli et al. (2011), Italy	Cohort study	79 patients with undiagnosed PE	• Diagnostic thoracocentesis • ELISA for VEGF levels measurement	1 Comparison of pleural fluid VEGF levels between patients with exudative and transudative PE 2 Comparison of pleural fluid VEGF levels between patients with MPE and benign exudative PE	• Selection **** • Comparability ** • Outcome *** *Overall* Fair quality
Hirayama et al. (2011), Japan	Cohort study	91 patients with undiagnosed PE	• Diagnostic thoracocentesis • Simultaneous blood sampling • ELISA for VEGF levels measurement	1 Comparison of pleural fluid and serum VEGF levels among patients with MPE, BPE and PE due to malignant mesothelioma 2 Correlation between pleural fluid and serum VEGF levels among patients with PE due to malignant mesothelioma	• Selection **** • Comparability ** • Outcome *** *Overall* Fair quality
Qian et al. (2012), China	Cohort study	103 patients with undiagnosed PE	• Diagnostic thoracocentesis • Simultaneous blood sampling • ELISA for VEGF levels measurement	Comparison of pleural fluid and serum VEGF levels between patients with MPE and tuberculous PE	• Selection **** • Comparability ** • Outcome *** *Overall* Fair quality
Zhang et al. (2012), China	Cohort study	102 patients with undiagnosed PE	• Diagnostic thoracocentesis • Simultaneous blood sampling • ELISA for VEGF levels measurement	Comparison of pleural fluid and serum VEGF levels between patients with MPE and BPE	• Selection **** • Comparability ‐ • Outcome *** *Overall* Low quality
Lieser et al. (2013), USA	Cohort study	19 patients with undiagnosed PE	• Diagnostic thoracocentesis • ELISA for VEGF levels measurement	Comparison of pleural fluid VEGF levels between patients with MPE and BPE	• Selection **** • Comparability ‐ • Outcome *** *Overall* Low quality

PE, pleural effusion; ELISA, enzyme‐linked immunosorbent assay; VEGF, vascular endothelial growth factor; MPE, malignant pleural effusion; BPE, benign pleural effusion.

### Meta‐analysis

The mean and standard deviation of the PF‐VEGF and S‐VEGF levels were used in order to compute the SMD. The calculation of the aforementioned summary effect requires knowing the individual study's mean and standard deviation (Higgins and Green 2008; Chen et al. 2015). The median can be very similar to the mean when the data are normally distributed, and this case occasionally can be used directly in meta‐analyses (Higgins and Green 2008). However, medians are usually reported when the data are skewed, and in this case, means and medians can be very different from each other (Higgins and Green 2008). Eight studies reported the medians of the PF‐VEGF and/or S‐VEGF, since the VEGF concentrations were not normally distributed and thus these studies were excluded from the statistical analysis (Thickett et al. 1999; Lim et al. 2000; Ishimoto et al. 2002; Tomimoto et al. 2007; Duysinx et al. 2008; Economidou et al. 2008; Fiorelli et al. 2011; Lieser et al. 2013). Two groups of patients were compared: patients with MPE (due to malignancies such as lung cancer, malignant mesothelioma, or metastatic cancer) and patients with BPE (due to benign conditions such as congestive heart failure, infection, or liver cirrhosis).

#### PF‐VEGF levels

Eleven out of 20 studies with a total number of 1126 patients were eligible for the mean difference analysis (Table 2) (Momi et al. 2002; Hamed et al. 2004; Jin et al. 2004; Sack et al. 2005; Daniil et al. 2007; Shu et al. 2007; Xue et al. 2007; Zhou et al. 2009; Hirayama et al. 2011; Qian et al. 2012; Zhang et al. 2012). One study was excluded since it provided the mean and standard deviation only for the lung cancer group of patients and not for the other malignancies group (Wells et al. 2013). All studies showed statistically significant higher PF‐VEGF levels among patients with MPE, except for one (Daniil et al. 2007).

**Table 2:** Data from the studies that compared pleural fluid VEGF levels between groups of patients and were included in the meta‐analysis

Study	MPE Number of patients PF‐VEGF (ng/mL)^a^	BPE Number of patients PF‐VEGF (ng/mL)^a^
Momi et al. (2002)	*n* = 38 2.01 ± 1.30	*n* = 89 0.50 ± 0.50
Jin et al. (2004)	*n* = 40 2.60 ± 0.72	*n* = 43 0.72 ± 0.58
Hamed et al. (2004)	*n* = 15 1.28 ± 0.28	*n* = 25 0.53 ± 0.41
Sack et al. (2005)	*n* = 96 2.28 ± 2.8	*n* = 118 0.64 ± 1.12
Shu et al. (2007)	*n* = 32 1.36 ± 1.49	*n* = 49 0.42 ± 0.32
Daniil et al. (2007)	*n* = 45 1.26 ± 1.26	*n* = 27 1.01 ± 0.97
Xue et al. (2007)	*n* = 42 1.14 ± 0.32	*n* = 45 0.68 ± 0.22
Zou et al. (2012)	*n* = 62 3.71 ± 1.65	*n* = 64 0.86 ± 0.37
Hirayama et al. (2011)	*n* = 66 4.43 ± 2.27	*n* = 25 1.17 ± 1.21
Zhang et al. (2012)	*n* = 52 0.36 ± 0.13	*n* = 50 0.12 ± 0.09
Qian et al. (2012)	*n* = 79 6.30 ± 1.14	*n* = 24 1.13 ± 0.35

PF‐VEGF, pleural fluid vascular endothelial growth factor; MPE, malignant pleural effusion; BPE, benign pleural effusion.

a VEGF levels are presented as mean ± standard deviation.

**Figure 2:**
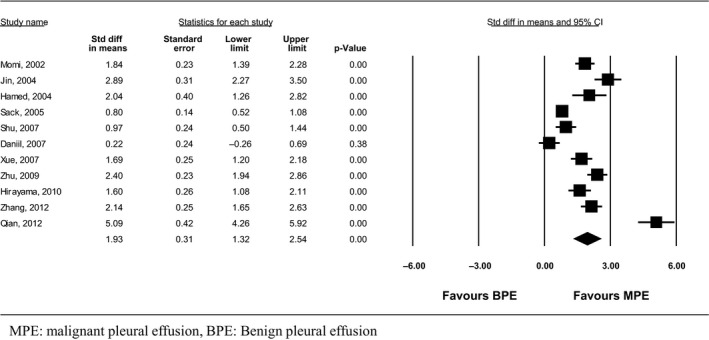
Standardized mean difference of pleural fluid vascular endothelial growth factor levels between patients with malignant pleural effusion (MPE) and benign pleural effusion (BPE).

The meta‐analysis showed that the PF‐VEGF levels in the MPE group were increased by 1.93 ng/mL compared to the BPE group (95% CI: 1.32–2.54, *Q* = 173, df (*Q*):10, *I* ^2^ = 94.2%, *P* < 0.05) (Fig. 2).

#### S‐VEGF levels

Seven out of 20 studies that evaluated 750 patients were eligible for the mean difference analysis (Table 3) (Momi et al. 2002; Hamed et al. 2004; Jin et al. 2004; Sack et al. 2005; Shu et al. 2007; Qian et al. 2012; Zhang et al. 2012). One study was excluded since it provided data for the S‐VEGF levels only for the malignant mesothelioma group of patients (Hirayama et al. 2011). All studies showed statistically significant higher S‐VEGF levels among patients with MPE, except for one (Sack et al. 2005).

**Table 3:** Data from the studies that compared serum VEGF levels between groups of patients and were included in the meta‐analysis

Study	MPE Number of patients S‐VEGF (ng/mL)^a^	BPE Number of patients S‐VEGF (ng/mL)^a^
Momi et al. (2002)	*n* = 38 1.19 ± 0.73	*n* = 89 0.34 ± 0.33
Jin et al. (2004)	*n* = 40 1.64 ± 0.62	*n* = 43 0.78 ± 0.50
Hamed et al. (2004)	*n* = 15 1.02 ± 0.24	*n* = 25 0.52 ± 0.15
Sack et al. (2005)	*n* = 96 0.46 ± 0.37	*n* = 118 0.45 ± 0.42
Shu et al. (2007)	*n* = 32 0.65 ± 0.53	*n* = 49 0.14 ± 0.28
Zhang et al. (2012)	*n* = 52 0.14 ± 0.12	*n* = 50 0.05 ± 0.05
Qian et al. (2012)	*n* = 79 0.16 ± 0.02	*n* = 24 0.06 ± 0.01

S‐VEGF, serum vascular endothelial growth factor; MPE, malignant pleural effusion; BPE, benign pleural effusion.

a VEGF levels are presented as mean ± standard deviation.

**Figure 3:**
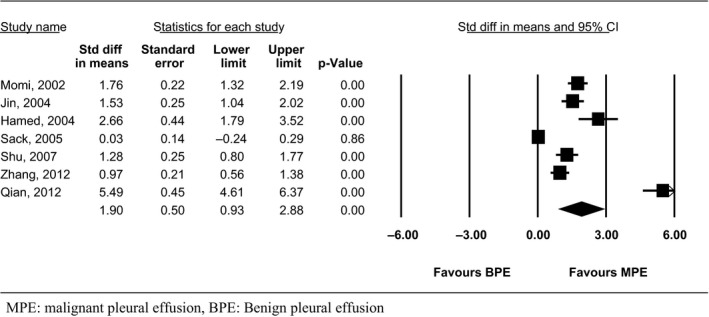
Standardized mean difference of serum vascular endothelial growth factor levels between patients with malignant pleural effusion (MPE) and benign pleural effusion (BPE).

The meta‐analysis showed that the S‐VEGF levels in the MPE group were increased by 1.90 ng/mL compared to the BPE group (95% CI: 0.93–2.88, *Q* = 173, df (*Q*): 6, *I* ^2^ = 96.7%, *P* < 0.05) (Fig. 3).

#### Publication bias

**Figure 4:**
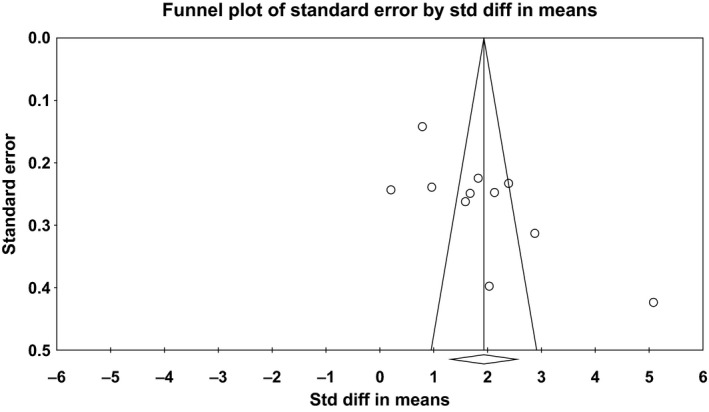
Funnel plot of pleural fluid vascular endothelial growth factor levels.

The funnel plot regarding PF‐VEGF levels seemed asymmetrical with studies missing at the bottom and some studies presenting as outliers with markedly different effect estimates (Fig. 4). This subjective visual impression was confirmed by the Egger's test (*P* < 0.05). Rosenthal's fail‐safe *N* was 1522, suggesting that a great number of studies (which could not have been missed by the present review) would be needed in order for the summary effect to become statistically nonsignificant. Finally, the Duval and Tweedie's Trim and Fill test showed that the “corrected” summary effect after the bias is removed remains unchanged.

**Figure 5:**
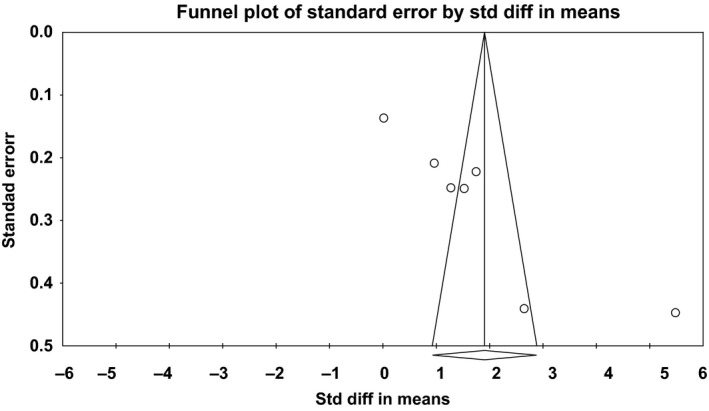
Funnel plot of serum vascular endothelial growth factor levels.

Concerning S‐VEGF levels, the funnel plot was notably asymmetrical (Fig. 5), Egger's test yielded a *P* < 0.05, Rosenthal's fail‐safe *N* was 459, and the trim and fill method computed an identical summary effect as our analysis. However, since there were only seven studies examining S‐VEGF levels, the power of the tests is reported to be low to distinguish chance from real asymmetry (Higgins and Green 2008).

## Discussion

The diagnostic evaluation of PE begins with clinical history and examination and thoracocentesis with pleural fluid analysis. However, pleural fluid analysis can misclassify transudates as exudative effusions approximately in 25% of the cases, while for 15% of patients diagnosis is never established (Porcel and Light 2006; Janda and Swiston 2010). Therefore, establishing reliable biomarkers may aid and improve the accuracy of PE diagnosis.

Vascular endothelial growth factor plays a critical role in angiogenesis, which is essential for tumor growth and metastasis (Bradshaw et al. 2013). Therefore, in a variety of malignancies, VEGF is overexpressed and thus the PF‐VEGF and/or S‐VEGF levels in MPE may be increased compared to BPE (Shu et al. 2007; Bradshaw et al. 2013). Current data strongly suggest that VEGF may act as a critical mediator in the pathogenesis of MPE, however, the exact mechanisms are still under investigation (Bradshaw et al. 2013). Since the late 1990s, it has been reported that PF‐VEGF levels vary depending on the cause of the PE (Cheng et al. 1999; Thickett et al. 1999; Yanagawa et al. 1999). More specifically, Thickett et al. (1999) reported higher PF‐VEGF levels among patients with MPE as compared to those with BPE. However, much overlap was reported between both PF‐VEGF and S‐VEGF levels in the various groups of patients (Chen et al. 2015).

Studies that followed reported similar findings. Namely, PF‐VEGF is present in both BPE and MPE; however, in the majority of the studies, its levels are reported to be statistically significantly increased in MPE (Lim et al. 2000; Tomimoto et al. 2007; Duysinx et al. 2008; Fiorelli et al. 2011). Furthermore, several studies that evaluated S‐VEGF levels reached the conclusion that VEGF is increased in the group of patients with MPE (Ishimoto et al. 2002; Momi et al. 2002; Hamed et al. 2004; Jin et al. 2004). A recent diagnostic accuracy meta‐analysis concluded that the maximum joint sensitivity and specificity of PF‐VEGF as a diagnostic biomarker is 0.75, suggesting that although its diagnostic value is not currently satisfactory, it may play a role in the diagnosis of MPE (Shen et al. 2012).

Our systematic review retrieved 20, mainly cohort, studies conducted during a 15‐year period. All of them examined PF‐VEGF and/or S‐VEGF levels in patients with undiagnosed PE, confirmed the diagnosis with all the relevant gold clinical standards and then compared the levels across groups of patients with different causes of PE. The majority of the studies showed that MPE was associated with higher PF‐VEGF and S‐VEGF levels and our meta‐analysis confirmed the findings of the primary studies. Namely, the levels of pleural and serum VEGF were significantly higher among patients with MPE compared to patients with BPE. As mentioned before, a diagnostic accuracy meta‐analysis concerning PF‐VEGF levels has already been conducted by Shen et al. (2012). A diagnostic accuracy meta‐analysis concerning serum VEGF levels in order to assess a summary specificity and sensitivity could not be performed with the present data since there were only two studies examining the sensitivity and specificity of the biomarker (Shu et al. 2007; Zhang et al. 2012). However, since the results of our study demonstrate that there is a difference in serum VEGF levels between BPE and MPE future studies that would also assess the sensitivity and specificity of this biomarker are needed.

This study has several limitations. First of all, the number of the studies that were eligible for the meta‐analysis and the corresponding sample size were relatively small, especially concerning the levels of S‐VEGF. Furthermore, half of the studies did not compare or adjust for any other characteristics, such as age, sex, or smoking status between the various groups of patients and thus they were classified as low‐quality studies. Despite this fact, these studies provided valuable information and thus we decided to include them in the meta‐analysis acknowledging that the quality of the combined estimate may also be affected.

Moreover, concerning the PF‐VEGF or S‐VEGF levels, up until now, there are no established thresholds. Therefore, there was much variation as well as significant overlap among PF‐VEGF or S‐VEGF levels in different groups of patients. Namely, the mean levels of PF‐VEGF between patients with MPE and BPE in the studies that were included in the meta‐analysis varied from 0.36 to 6.3 ng/mL and 0.12 to 1.17 ng/mL, respectively. Accordingly, S‐VEGF levels among patients with MPE and BPE varied from 0.14 to 1.64 ng/mL and 0.05 to 0.78 ng/mL, respectively. Thus, there was considerable variation among studies, and this was reflected in the significant proportion of heterogeneity in our meta‐analysis. Finally, language selection may have biased our results. There was evidence of publication bias, however, when applying the trim and fill method, the summary effect remained unaffected.

Despite these limitations, this study is the first systematic review and meta‐analysis of the studies comparing PF‐VEGF and/or S‐VEGF levels between patients presenting with MPE and BPE and thus its results may have clinical implications. Specifically, VEGF could potentially be a useful biomarker aiding in the differential diagnosis of patients presenting with undiagnosed PE and leading to better clinical management. Interestingly, aside from PF‐VEGF levels, which require thoracocentesis in order to be measured, we also found that S‐VEGF levels, which require the least invasive technique in order to be measured namely blood sampling, are increased in MPE. However, the number of studies retrieved from the systematic review that has focused on PF‐VEGF and/or S‐VEGF levels in PE is relatively small and thus further studies are necessary.

In conclusion, this study showed that VEGF may be a prominent biomarker in the differentiation between MPE and BPE. The results of this study could act as a basis for the development of further research in this field.

## Conflict of interest

None declared.

## Literature Search Strategy

### PubMed (MEDLINE)

We used the PubMed Advanced Search Builder and the filters Species (Humans) and Languages (English). The search was conducted between 1 and 7 of July, 2015.

1. (“vascular endothelial growth factor”) AND “pleural effusion”

Query translation: “vascular endothelial growth factor”[All Fields] AND “pleural effusion”[All Fields]

((“vascular endothelial growth factor”) AND “pleural effusion”) AND diagnosis

Query translation: (“vascular endothelial growth factor”[All Fields] AND “pleural effusion”[All Fields]) AND (“diagnosis”[Subheading] OR “diagnosis”[All Fields] OR “diagnosis”[MeSH Terms])

((“vascular endothelial growth factor”) AND “benign pleural effusion”) AND “malignant pleural effusion”

Query translation: (“vascular endothelial growth factor”[All Fields] AND “benign pleural effusion”[All Fields]) AND “malignant pleural effusion”[All Fields]

((“vascular endothelial growth factor”) AND “pleural effusion”) AND serum

Query translation: (“vascular endothelial growth factor”[All Fields] AND “pleural effusion”[All Fields]) AND (“serum”[MeSH Terms] OR “serum”[All Fields])

(((“vascular endothelial growth factor”) AND “pleural effusion”) AND serum) AND diagnosis

Query translation: ((“vascular endothelial growth factor”[All Fields] AND “pleural effusion”[All Fields]) AND (“serum”[MeSH Terms] OR “serum”[All Fields])) AND (“diagnosis”[Subheading] OR “diagnosis”[All Fields] OR “diagnosis”[MeSH Terms])

((“vascular endothelial growth factor”) AND “tuberculous pleurisy”) AND “malignant pleural effusion”

Query translation: (“vascular endothelial growth factor”[All Fields] AND “tuberculous pleurisy”[All Fields]) AND “malignant pleural effusion”[All Fields]

((“vascular endothelial growth factor”) AND “tuberculous pleurisy”) AND “lung cancer”

Query translation: (“vascular endothelial growth factor”[All Fields] AND “tuberculous pleurisy”[All Fields]) AND “lung cancer”[All Fields]

((“vascular endothelial growth factor”) AND “tuberculous pleurisy”) AND “metastatic cancer”

Query translation: (“vascular endothelial growth factor”[All Fields] AND “tuberculous pleurisy”[All Fields]) AND “metastatic cancer”[All Fields]

((“vascular endothelial growth factor”) AND “heart failure”) AND “malignant pleural effusion”

Query translation: (“vascular endothelial growth factor”[All Fields] AND “heart failure”[All Fields]) AND “malignant pleural effusion”[All Fields]

((“vascular endothelial growth factor”) AND “heart failure”) AND “lung cancer”

Query translation: (“vascular endothelial growth factor”[All Fields] AND “heart failure”[All Fields]) AND “lung cancer”[All Fields]

((“vascular endothelial growth factor”) AND “heart failure”) AND “metastatic cancer”

Query translation: (“vascular endothelial growth factor”[All Fields] AND “heart failure”[All Fields]) AND “metastatic cancer”[All Fields]

This search strategy retrieved 451 studies in total. By applying the aforementioned filters the number of the studies was limited to 344.

### Scopus (ELSEVIER)

We used the Advanced Search form and the filters Subject Area (Medicine) and Language (English). The search was conducted between 8 and 14 of July, 2015.

1. TITLE‐ABS‐KEY (”vascular endothelial growth factor”) AND TITLE‐ABS‐KEY (”pleural effusion”)
2. TITLE‐ABS‐KEY (”vascular endothelial growth factor”) AND TITLE‐ABS‐KEY (”pleural effusion”) AND TITLE‐ABS‐KEY (diagnosis)
3. TITLE‐ABS‐KEY (”vascular endothelial growth factor”) AND TITLE‐ABS‐KEY (”benign pleural effusion”) AND TITLE‐ABS‐KEY (”malignant pleural effusion”)
4. TITLE‐ABS‐KEY (”vascular endothelial growth factor”) AND TITLE‐ABS‐KEY (”pleural effusion”) AND TITLE‐ABS‐KEY (serum)
5. TITLE‐ABS‐KEY (”vascular endothelial growth factor”) AND TITLE‐ABS‐KEY (”pleural effusion”) AND TITLE‐ABS‐KEY (serum) AND TITLE‐ABS‐KEY (diagnosis)
6. TITLE‐ABS‐KEY (”vascular endothelial growth factor”) AND TITLE‐ABS‐KEY (”tuberculous pleurisy”) AND TITLE‐ABS‐KEY (”malignant pleural effusion”)
7. TITLE‐ABS‐KEY (”vascular endothelial growth factor”) AND TITLE‐ABS‐KEY (”tuberculous pleurisy”) AND TITLE‐ABS‐KEY (”lung cancer”)
8. TITLE‐ABS‐KEY (”vascular endothelial growth factor”) AND TITLE‐ABS‐KEY (”tuberculous pleurisy”) AND TITLE‐ABS‐KEY (”metastatic cancer”)
9. TITLE‐ABS‐KEY (”vascular endothelial growth factor”) AND TITLE‐ABS‐KEY (”heart failure”) AND TITLE‐ABS‐KEY (”malignant pleural effusion”)
10. TITLE‐ABS‐KEY (”vascular endothelial growth factor”) AND TITLE‐ABS‐KEY (”heart failure”) AND TITLE‐ABS‐KEY (”lung cancer”)
11. TITLE‐ABS‐KEY (”vascular endothelial growth factor”) AND TITLE‐ABS‐KEY (”heart failure”) AND TITLE‐ABS‐KEY (”metastatic cancer”)

This search strategy retrieved 458 studies in total. By applying the aforementioned filters the number of the studies was limited to 366.

### Cochrane Central Register of Controlled Trials (CENTRAL)

The search was conducted between 15 and 17 of July, 2015.

1. “vascular endothelial growth factor” AND “pleural effusion” in selected fields: Title, Abstract, Keywords
2. “vascular endothelial growth factor” AND “pleural effusion” AND diagnosis in selected fields: Title, Abstract, Keywords
3. “vascular endothelial growth factor” AND “benign pleural effusion” AND “malignant pleural effusion” in selected fields: Title, Abstract, Keywords
4. “vascular endothelial growth factor” AND “pleural effusion” AND “serum” in selected fields: Title, Abstract, Keywords
5. “vascular endothelial growth factor” AND “pleural effusion” AND serum AND diagnosis in selected fields: Title, Abstract, Keywords
6. “vascular endothelial growth factor” AND “tuberculous pleurisy” AND “malignant pleural effusion” in selected fields: Title, Abstract, Keywords
7. “vascular endothelial growth factor” AND “tuberculous pleurisy” AND “lung cancer” in selected fields: Title, Abstract, Keywords
8. “vascular endothelial growth factor” AND “tuberculous pleurisy” AND “metastatic cancer” in selected fields: Title, Abstract, Keywords
9. “vascular endothelial growth factor” AND “heart failure” AND “malignant pleural effusion” in selected fields: Title, Abstract, Keywords
10. “vascular endothelial growth factor” AND “heart failure” AND “lung cancer” in selected fields: Title, Abstract, Keywords
11. “vascular endothelial growth factor” AND “heart failure” AND “metastatic cancer” in selected fields: Title, Abstract, Keywords

This search strategy retrieved 10 studies in total.
